# Gli Phosphorylation Code in Hedgehog Signal Transduction

**DOI:** 10.3389/fcell.2022.846927

**Published:** 2022-01-25

**Authors:** Mengmeng Zhou, Jin Jiang

**Affiliations:** ^1^ Department of Molecular Biology, UT Southwestern Medical Center, Dallas, TX, United States; ^2^ Department of Pharmacology, UT Southwestern Medical Center, Dallas, TX, United States

**Keywords:** Hedgehog, Ci, Gli, Fu, Ulk3, PKA, GSK3, CK1

## Abstract

Hedgehog (Hh) family of secreted proteins governs many key processes in embryonic development and adult tissue homeostasis in species ranging from insects to human. Deregulation of Hh signaling has been implicated in a wide range of human diseases including birth defect and cancer. Hh signaling pathway culminates in the conversion of the latent transcription factor Cubitus interruptus (Ci)/Gli from a repressor form (Ci^R^/Gli^R^) into an activator form (Ci^A^/Gli^A^). Both the production of Ci^R^/Gli^R^ in the absence of Hh and the formation of Ci^A^/Gli^A^ in response to Hh are regulated by phosphorylation. Whereas previous studies demonstrated that sequential phosphorylation by protein kinase A (PKA), glycogen synthase kinase 3 (GSK3), and casein kinase 1 (CK1) at multiple Ser/Thr clusters in the C-terminal region of Ci/Gli targets it for proteolytic processing to generate Ci^R^/Gli^R^, recent studies revealed that phosphorylation of Ci/Gli by the Fused (Fu)/Unc-51 like kinase (Ulk) family kinases Fu/Ulk3/Stk36 and other kinases contributes to Ci/Gli activation. Fu/Ulk3/Stk36-mediated phosphorylation of Ci/Gli is stimulated by Hh, leading to altered interaction between Ci/Gli and the Hh pathway repressor Sufu. Here we review our current understanding of how various Ci/Gli phosphorylation events are regulated and how they influence Hh signal transduction.

## Introduction

**FIGURE 1:**
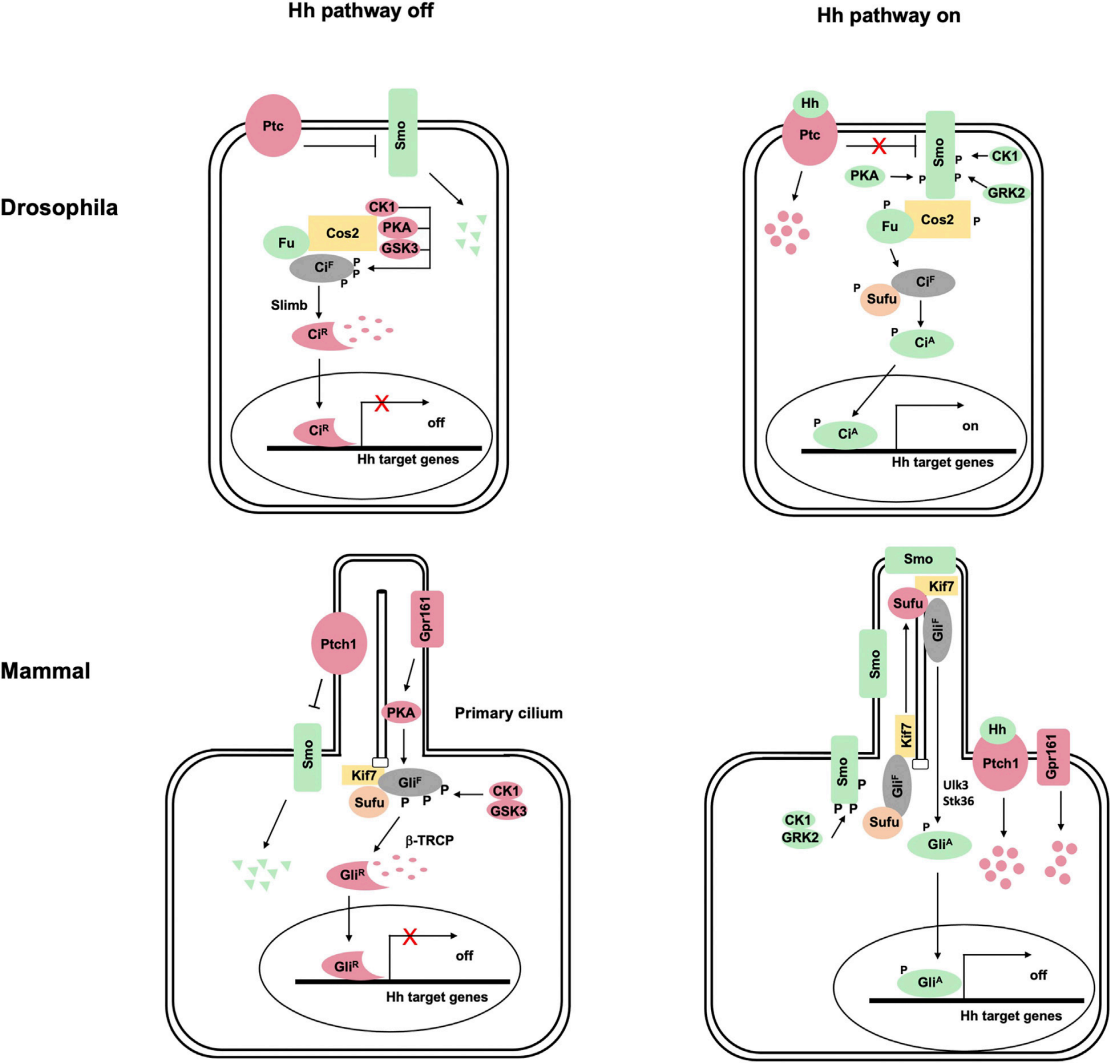
The hedgehog signaling transduction in *Drosophila* and vertebrates. **(A)** In *Drosophila*, the 12-span transmembrane protein Ptc represses the GPCR-family protein Smo in the absence of Hh ligand, leading to the degradation of Smo by the ubiquitylation pathway. Full-length Ci (Ci^F^) forms a complex with the kinesin-like protein Cos2 and the Ser/Thr kinase Fu, which recruits PKA, GSK3, and CK1 to phosphorylate Ci. Phosphorylation of Ci^F^ targets it for Slimb-mediated ubiquitination, followed by proteasome-mediated proteolysis to generate Ci^R^. Binding of Hh to Ptc releases its inhibition of Smo, allowing Smo to be phosphorylated by PKA, CK1 and GRK2. Activated Smo not only blocks Ci^F^ phosphorylation by PKA/GSK3/CK1 and thus the production of Ci^R^ but also converts Ci^F^ into the activator form (Ci^A^) through activating the Fu kinase, which directly phosphorylates Ci^F^ to release its inhibition by Sufu. **(B)** Vertebrate Hh signal transduction depends on primary cilia. In the absence of Hh, both Ptch1 and Gpr161 (a GPCR coupled to G⍺s) are localized in primary cilia where Ptch1 prevents Smo ciliary localization and Gpr161 activates PKA. Full-length Gli (Gli^F^; mainly Gli3 and Gli2) is phosphorylated by PKA, GSK3, and CK1, which targets it for β-TRCP-mediated ubiquitination, followed by proteasome-mediated proteolysis to generate Gli^R^. Binding of Hh to Ptch1 inhibits its activity and promotes its ciliary exit, allowing Smo to be phosphorylated by CK1 and GRK2 and accumulated in primary cilia. Activated Smo inhibits Ci phosphorylation by PKA and the production of Gli^R^ in part by promoting Gpr161 ciliary exit. In addition, activated Smo converts Gli^F^ into Gli^A^ at least in part through Ulk3 and STK36, which phosphorylate Gli^F^ to attenuate the inhibition by Sufu.

The Hedgehog (Hh) family of signaling molecules governs embryonic development and adult tissue homeostasis in species ranging from insects to mammals, and aberrant Hh signaling contributes to a wide range of human diseases (Nieuwenhuis and Hui, 2005; Jiang and Hui, 2008; Petrova and Joyner, 2014; Jiang, 2021). The Hh signal is transduced via a conserved pathway culminating in the conversion of the latent transcription factor Ci/Gli from a repressor (Ci^R^/Gli^R^) into an activator (Ci^A^/Gli^A^) (Figure 1) (Wilson and Chuang, 2010; Jiang, 2021). In the absence of Hh, the twelve-span transmembrane protein Patched (Ptc) inhibits the GPCR family member Smoothened (Smo), allowing full-length Ci/Gli (Ci^F^/Gli^F^) to be proteolytically processed to generate Ci^R^/Gli^R^ that lacks its C-terminal coactivator binding domain but retains its N-terminal corepressor binding domain (Hui and Angers, 2011; Chen and Jiang, 2013). Binding of Hh to Ptc alleviates its inhibition of Smo, allowing Smo to signal intracellularly to block Ci^R^/Gli^R^ production and convert Ci^F^/Gli^F^ into Ci^A^/Gli^A^ (Qi and Li, 2020).

Vertebrates have three Gli family members: Gli1, Gli2, and Gli3 (Hui and Angers, 2011). Both Gli2 and Gli3 can be proteolytically processed to generate Gli^R^ in signaling off state and converted into Gli^A^ upon Hh stimulating. Gli1 functions exclusively as transcriptional activator and its expression is induced by Hh signaling, forming a positive feedback loop to amplify Hh pathway outputs.

## Regulation of Ci/Gli Processing by Multi-Site Phosphorylation

Genetic studies in *Drosophila* identified three kinases, PKA, GSK3, and CK1, as well as an E3 ubiquitin ligase component Slimb as essential for Ci processing into Ci^R^ (Figure 1) (Jiang and Struhl, 1995; Li et al., 1995; Jiang and Struhl, 1998; Jia et al., 2002; Price and Kalderon, 2002; Jia et al., 2005). Subsequent biochemical experiments demonstrated that these kinases sequentially phosphorylate Ci at three phosphorylation S/T clusters in its C-terminal half, with PKA phosphorylating Ci on S838, S856, and S892, priming its further phosphorylation by GSK3 and CK1 on adjacent S/T residues to generate a Slimb binding site that recruits an E3 ubiquitin ligase complex SCF^Slimb^ to target Ci for ubiquitination, followed by proteasome-mediated proteolysis to generate Ci^R^ (Aza-Blanc et al., 1997; Price and Kalderon, 1999; Wang et al., 1999; Price and Kalderon, 2002; Jia et al., 2002; Jia et al., 2005; Smelkinson et al., 2007; Zhang and Jiang, 2021). Ci^R^ lacks the C-terminal CBP binding domain but retains the N-terminal co-repressor binding domain (Akimaru et al., 1997; Zhang et al., 2013), and actively inhibits the expression of a subset of Hh target genes (Jiang and Struhl, 1998; Methot and Basler, 1999).

**FIGURE 2:**
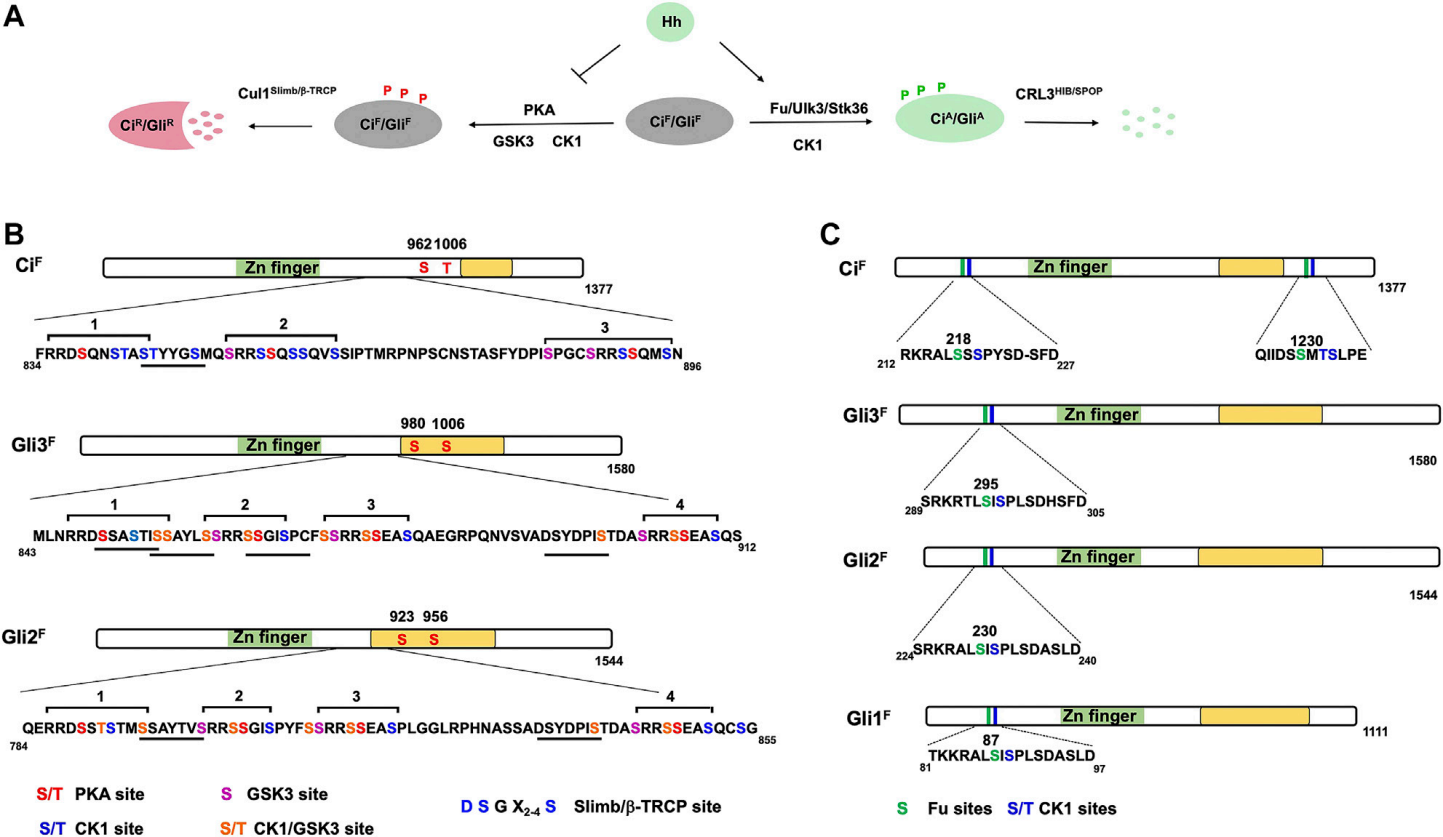
Ci/Gli phosphorylation code. **(A)** Ci/Gli is phosphorylated on different sites by distinct sets of kinases depending on the Hh signaling states. In the absence of Hh, phosphorylation of Ci^F^/Gli^F^ by PKA/CK1/GSK3 negatively regulates Hh signaling by converting Ci^F^/Gli^F^ into Ci^R^/Gli^R^ whereas in the presence of Hh, phosphorylation of Ci^F^/Gli^F^ by Fu/Ulk3/Stk36 plays a positive role by promoting the formation of Ci^A^/Gli^A^. **(B–C)** Schematic representation of Ci and Gli showing the PKA/CK1/GSK3 phosphorylation sites **(B)** and Fu/Ulk3/Stk36 phosphorylation sites **(C)**. Individual kinase sites are color coded. The underlines indicate the Slimb/β-TRCP binding sites in Ci/Gli. The green box represents the Zn-finger DNA-binding domain whereas the yellow box indicates the transactivation domain.

The molecular mechanism underlying Ci processing appears to be conserved for Gli proteins as revealed initially by genetic study in *Drosophila* (Aza-Blanc et al., 2000). Subsequent biochemical studies demonstrated that sequential phosphorylation by PKA, GSK3 and CK1 at four phosphorylation clusters in the C-terminal half of Gli2 and Gli3 generate multiple degrons that recruits β-TRCP, the vertebrate ortholog of Slimb (Figure 2) (Wang et al., 2000; Bhatia et al., 2006; Pan et al., 2006; Tempe et al., 2006; Wang and Li, 2006; Wen et al., 2010). In contrast to Gli3 where β-TRCP-mediated proteolysis mainly leads to partial degradation and therefore the production of Gli^R^, Gli2 proteolysis often leads to complete degradation of the protein (Pan et al., 2006; Pan and Wang, 2007), which may explain why Gli^R^ is mainly contributed by Gli3 (Hui and Angers, 2011).

## Processing-Independent Inhibition of Ci/Gli by PKA

An early study suggested that PKA not only regulates the production of Ci^R^ but also inhibits Ci^A^ because blockage of Ci^R^ production by *slimb* mutation in *Drosophila* wing imaginal discs only resulted in ectopic expression of *decapentaplegic* (*dpp*), a Hh target gene normally inhibited by Ci^R^; however, inactivation of PKA not only resulted in ectopic expression of *dpp* but also *ptc*, which is normally activated by Ci^A^ (Jiang and Struhl, 1998; Methot and Basler, 1999; Wang et al., 1999). Furthermore, processing-deficient forms of Ci is still inhibited by PKA (Wang et al., 1999; Marks and Kalderon, 2011; Little et al., 2020). In addition to phosphorylating the three phosphorylation clusters in the C-terminal half of Ci, PKA also phosphorylates two additional sites in the C-terminal half of Ci (Figure 2). While mutating the PKA sites in the first three phosphorylation clusters (Ci^−PKAm3^) is sufficient to block Ci processing, mutating all five PKA sites generated a more active Ci variant (Ci^−PKAm5^) than Ci^−PKAm3^ (Price and Kalderon, 1999), suggesting that phosphorylation of the C-terminal two PKA sites may inhibit Ci^A^ activity.

Studies in mammalian systems also demonstrated that PKA plays a critical role in restricting the activator activity of Gli2. *PKA* null mice exhibited neural tube phenotypes indistinguishable from those of *Ptc* ^*−/−*^ mice, with full-blown ectopic Hh pathway activation. Removal of Gli2 from *PKA* null mice suppressed the ectopic expression of the ventral markers (Tuson et al., 2011). Furthermore, Gli2 was accumulated at the tips of primary cilia in *PKA* null MEF cells in the absence of Hh (Tuson et al., 2011). A subsequent study identified two PKA sites in Gli2/3 whose mutations resulted in increased Gli^A^ activity (Figure 2) (Niewiadomski et al., 2014). However, it remains undetermined how phosphorylation of Ci/Gli by PKA inhibits its activator activity. PKA may also phosphorylate other substrates to restrict Ci/Gli activity. For example, phosphorylation of Sufu by PKA increased Sufu abundance in mammalian cells (Chen et al., 2011), which could account for the inhibition of Gli^A^.

## Hh Inhibits Ci/Gli Processing by Regulating Its Phosphorylation

Hh signaling inhibits Ci/Gli processing to prevent the production of Ci^R^/Gli^R^. Using mobility shift as a readout for Ci phosphorylation, an early study revealed that Hh signaling reduced the overall levels of Ci phosphorylation (Chen et al., 1999). Using phospho-specific antibodies that recognized phosphorylated PKA sites in Ci or Gli2/3, two later studies showed that Hh inhibited Ci phosphorylation in wing imaginal discs and Gli2/3 phosphorylation in primary cilia (Zhang et al., 2005; Li et al., 2017). Another study using quantitative mass spec showed that Hh inhibits Gli2 phosphorylation at multiple sites (Niewiadomski et al., 2014). Then the question becomes how Hh signaling inhibits Ci/Gli phosphorylation.

### Regulation of Ci/Gli Phosphorylation by Protein Complexes

In *Drosophila*, Ci phosphorylation and processing are facilitated by a protein complex consisting of a kinesin-like protein Costal2 (Cos2) and a Ser/Thr kinase Fused (Fu), mutations of which resulted in Ci processing defect (Sisson et al., 1997; Zhou and Kalderon, 2011). In the absence of Hh, Cos2/Fu simultaneously binds Ci and its kinases, including PKA, GSK3, and CK1, and acts as a molecular scaffold to bring kinases and substrate in close proximity to facilitate Ci phosphorylation (Zhang et al., 2005). Hh signaling causes disassembly of the Ci-Cos2/Fu-kinase complex at least in part through interaction between Smo C-terminal intracellular tail and Cos2 (Jia et al., 2003; Zhang et al., 2005). Hh also induces Cos2 phosphorylation by Fu to inhibit the association between Ci and Cos2/Fu, which could contribute to the disassembly of the processing complex (Ruel et al., 2007). However, a later study revealed that mutating the Fu phosphorylation sites in Cos2 did not have a discernable effect on Hh pathway activity *in vivo* (Zhou and Kalderon, 2011; Zadorozny et al., 2015), suggesting that other mechanism(s) such as dissociation of kinases from the processing complex or sequestration of kinase away from Ci (see below) may be sufficient to block Ci phosphorylation and processing.

Kif7, the mammalian homolog of Cos2, is required for efficient Gli3 processing but the underlying mechanism remains undetermined (Cheung et al., 2009; Endoh-Yamagami et al., 2009; Liem et al., 2009). Mammalian Sufu is critical for Gli processing to generate Gli^R^ in addition to its role in inhibiting Gli^A^, which explains why loss of Sufu in mammals resulted in robust Hh pathway activation (Chen et al., 2009; Humke et al., 2010; Wang et al., 2010). Sufu forms a complex with both GSK3 and Gli3 to facilitate Gli3 phosphorylation by GSK3 (Kise et al., 2009).

### Regulation of Ciliary PKA Activity by Modulating GPCR and AC

In the vertebrate Hh pathway, the production of Gli^R^ depends on primary cilia because Gli processing is impeded when ciliogenesis is affected (Bangs and Anderson, 2017). Consistent with this, both Kif7 and Sufu/Gli are found in or transit through the primary cilia (Chen et al., 2009; Cheung et al., 2009; Endoh-Yamagami et al., 2009; Liem et al., 2009; Tukachinsky et al., 2010). In addition, the key regulatory components of Gli phosphorylation and processing including PKA holoenzyme and proteosome are enriched at the ciliary base (Wigley et al., 1999; Barzi et al., 2010; Tuson et al., 2011; Mick et al., 2015). Smo is a class F GPCR coupled to Gαi (Riobo et al., 2006; Ogden et al., 2008; Qi et al., 2019); however, whether Smo blocks Ci/Gli phosphorylation though Gαi to downregulate cAMP-dependent PKA activity has remained controversial (Ogden et al., 2008; Praktiknjo et al., 2018). Gpr161, a GPCR coupled to Gαs, is localized in the primary cilia in quiescent cells, which is thought be responsible for the local production of cAMP for PKA activation (Mukhopadhyay et al., 2013). Indeed, Gli2/3 processing was blocked in Gpr161 mutant mouse embryos, leading to constitutive Hh pathway activation and ventralization of neural tubes (Mukhopadhyay et al., 2013). In response to Hh stimulation, Gpr161 exits primary cilia through binding to β-arrestin (Mukhopadhyay et al., 2013; Pal et al., 2016). Gpr161 is not the only GPCR implicated in the regulation of PKA and Hh signaling. Another ciliary localized orphan GPCR, Gpr175, positively regulates Hh signaling by decreasing Gli^R^ levels through Gαi (Singh et al., 2015). However, whether loss of Gpr161 from the primary cilia or increasing Gpr175 activity leads to reduction of cAMP in cilia has not been directly tested.

Using optogenetic and chemogenetic tools to control the activity of GPCR or adenylyl cyclase (AC) and thus the subcellular location for cAMP production, a recent study demonstrated that ciliary but not cytoplasmic production of cAMP can inhibit Hh signaling through activating a ciliary pool of PKA (Truong et al., 2021). Consistent with the notion that Hh pathway activity is regulated by local production of cAMP at primary cilia, an early study using targeted cAMP sensor to measure local cAMP concentration found that basal ciliary cAMP is fivefold higher than whole-cell cAMP (Moore et al., 2016). This study also found that the elevated basal ciliary cAMP level is due to increased AC5/6 activity induced by PIP3 and that Shh reduces ciliary cAMP levels by promoting calcium influx to inhibit AC5/6 (Moore et al., 2016). However, by employing biosensors optimized for ciliary cAMP and strategies to separate ciliary signals from whole cell body signals, a later study found that ciliary cAMP was not elevated compared to cellular cAMP and that ciliary cAMP levels remained unchanged after Hh stimulation (Jiang et al., 2019).

### Inhibition of Ci/Gli Phosphorylation via Sequestration of PKA by Smo

Regardless of whether Hh regulates ciliary cAMP levels, cAMP-independent mechanisms could be involved to regulate Ci/Gli phosphorylation by PKA. Indeed, early studies in *Drosophila* showed that a cAMP-independent and constitutively mouse PKA catalytic subunit (PKAc) can fully rescue Hh signaling defects in *PKA* mutants (Jiang and Struhl, 1995; Li et al., 1995). In addition, Cos2 can recruit PKAc to phosphorylate Ci, which is antagonized by Hh signaling (Zhang et al., 2005). However, it remains unclear whether Kif7 can promote Gli phosphorylation by PKA. Using biosensors for measuring cytosolic or membrane associated PKA activity, it has been shown that Hh increased PKA activity localized on the plasm membrane without changing the overall cytoplasmic PKA activity *in Drosophila*, and that the increased PKA activity on plasm membrane was due to stabilization of PKAc by Smo (Li et al., 2014). Furthermore, Hh induced the formation of a Smo/PKAc complex to promote Smo phosphorylation on one hand and sequester PKAc away from Ci on the other hand (Li et al*.,* 2014; Ranieri et al., 2014). A recent study showed that phosphorylation of Smo C-tail by GRK2 induced the formation of a Smo/PKAc complex to prevent PKAc from phosphorylating Gli in mammalian cells (Arveseth et al., 2021), suggesting that sequestration of PKAc by Smo could be a conserved mechanism by which activated Smo inhibit Ci/Gli phosphorylation.

## Regulation of Ci^A^/Gli^A^ by Fu Family Kinases

Blocking Ci/Gli processing is insufficient to convert Ci^F^/Gli^F^ into Ci^A^/Gli^A^ because Sufu binds Ci/Gli to inhibit its transcriptional activator activity (Shi et al., 2014a; Han et al., 2015). In *Drosophila*, the Ser/Thr kinase Fu is required for high levels of Hh to convert Ci into labile Ci^A^ by antagonizing Sufu (Ohlmeyer and Kalderon, 1998). In response to Hh, Smo C-tail undergoes a conformational change that exposes a Cos2 binding domain to recruit Cos2/Fu, and dimerization/oligomerization of Smo C-tail causes clustering of Cos2/Fu to induce Fu autophosphorylation and activation (Zhao et al., 2007; Shi et al., 2011; Zhang et al., 2011; Zhou and Kalderon, 2011).

### Ci Phosphorylation by Fu Contributes to Ci Activation

Although Hh stimulates phosphorylation of Sufu through Fu (Lum et al., 2003), mutating the phosphorylation sites on Sufu does not affect its ability to inhibit Ci or its inhibition by Hh both in cultured cells and in wing imaginal discs (Zhou and Kalderon, 2011; Han et al., 2019), suggesting that Fu activates Ci by phosphorylating another substrate(s). A recent study demonstrated that Fu directly phosphorylates Ci on Ser218 and Ser1230, which primes CK1-mediated phosphorylation on their adjacent sites, and that these phosphorylation events contribute to Ci activation (Figure 2) (Han et al., 2019). Sequence alignment of the newly identified sites on Ci and a previous identified sites on Cos2 revealed a Fu phosphorylation consensus sequence: S/T(X)_5_D/E (Figure 2). Using phospho-specific antibodies, Han et al. (2019) showed that phosphorylation of Ci at S218 and S1230 is stimulated by Hh but suppressed by Sufu. Phospho-mimetic mutations of the two Fu sites and adjacent CK1 sites on Ci attenuated Sufu binding while increased the binding of Transportin (Trn) and the transcriptional coactivator CBP, resulting in Ci activation (Han et al., 2019). Interestingly, the phosphorylation levels at Fu sites increased progressively in response to increasing doses of Hh, which correlated with gradual change in pathway activities (Han et al., 2019), suggesting that Hh signaling gradient is translated into a Ci phosphorylation and activity gradient.

### Regulation of Gli^A^ by Ulk3/Stk36

Although knockout the Fu homolog Stk36 (mFu) in mice did not affect Hh signaling during development (Chen et al., 2005; Merchant et al., 2005), the presence of another Fu related kinase Ulk3 raised the possibility that Ulk3 and Stk36 may act redundantly (Maloverjan et al., 2010). Indeed, combined depletion of Ulk3 and Stk36 resulted in more dramatic reduction of Hh pathway activity as well as Smo-driven medulloblastoma growth than depletion of either Ulk3 or Stk36 alone (Han et al., 2019). Sequence alignment showed that the Ci N-terminal phosphorylation clusters, corresponding to S230/S232 in mouse Gli2, are highly conserved among three Gli proteins in mammals and that S230 conforms to the Fu consensus site (Figure 2). Mutating S230/S232 to Ala reduced Hh-induced Gli2 activity whereas converting them to Asp to mimic phosphorylation increased Gli2 activity by reducing the binding of Sufu (Han et al., 2019). Using a phospho-specific antibody that recognizes this phosphorylation cluster, Han et al. (2019) showed that S230/S232 phosphorylation is induced by Hh or Ulk3 overexpression and that Hh-induced S230/S232 phosphorylation is diminished by combined depletion of Ulk3 and Stk36. Consistent with Gli being activated in primary cilia, blocking Gli2 ciliary localization by depleting Kapβ abolished Hh-induced phosphorylation at S230/S232 (Han et al., 2017; Han et al., 2019). Interestingly, the levels of phosphorylation at S230/232 increased progressively in response to increasing levels of Shh, which correlated with gradually increased Hh pathway activity as measured by the *Gli-luc* reporter assay (Han et al., 2019).

## Regulation of Ci/Gli by Other Phosphorylation Events

In addition to the opposing phosphorylation events mediated by two distinct sets of kinases that regulate the production of Ci^R^/Gli^R^ and Ci^A^/Gli^A^, respectively, several studies have revealed that Ci/Gli is regulated by other phosphorylation events. For example, one study showed that phosphorylation of Ci/Gli by CK1 at multiple S/T residues located in the HIB/SPOP-binding motifs attenuates HIB/SPOP-mediated ubiquitination and degradation of Ci^A^/Gli^A^, thus preventing premature termination of Hh pathway activity (Shi et al., 2014b). In basal cell carcinomas (BCCs), centrosome-associated aPKC functions as a positive regulator of Hh signaling by phosphorylating Gli1 to increase its DNA binding activity (Atwood et al., 2013). In esophageal cancer, mTOR signaling promotes Hh signaling through S6K1-mediated Gli1 phosphorylation at Ser84, which releases Gli1 from its repressor Sufu (Wang et al., 2012). Polo-like kinase-1 (Plk1), a critical cell cycle regulator, phosphorylates Gli1 at S481 to increase its nuclear export and binding to Sufu, leading to attenuated Hh signaling activity (Zhang et al., 2019). Consistent with a previous finding that CK2 promotes Hh signaling by phosphorylating Ci/Gli in addition to Smo (Jia et al., 2010), a recent phosphoproteomics study identified CK2 as critical for the stabilization and transcriptional activity of Gli2 in granule neuron precursors and showed that pharmacological inhibition of CK2 attenuated the growth of Shh-type medulloblastoma cells expressing a drug resistant Smo mutant (Purzner et al., 2018).

## Conclusion

Although many kinases and phosphorylation events are involved in the regulation of Ci/Gli activity, how these phosphorylation events are regulated by Hh signaling is not fully understood. For example, how is Ulk3/Stk36 activated by Hh? Does Ulk3/Stk36 phosphorylate Gli in primary cilia? Are there more Fu/Ulk3/Stk36 sites in Ci/Gli whose phosphorylation contributes Ci/Gli activation? It is possible that Ci/Gli is regulated by additional phosphorylation events that need to be discovered. In addition, Gli proteins could be activated in cancer cells by oncogenic pathways independent of the canonical Hh pathway (Pietrobono et al., 2019). Full understanding of canonical and non-canonical activation of Ci/Gli by phosphorylation may provide new strategies for cancer drug development.
